# Diphenolic acid-modified PAMAM/chlorinated butyl rubber nanocomposites with superior mechanical, damping, and self-healing properties

**DOI:** 10.1080/14686996.2020.1861912

**Published:** 2021-01-22

**Authors:** Yao Lu, Jincheng Wang, Le Wang, Shiqiang Song

**Affiliations:** College of Chemistry and Chemical Engineering, Shanghai University of Engineering Science, Shanghai, P.R.China

**Keywords:** PAMAM, multiple hydrogen bonds, rubber, damping, self-healing, 103 Composites, 212 Surface and interfaces, 503 TEM, STEM, SEM

## Abstract

Based on its excellent damping properties, traditional rubber has been widely used in various industries, including aerospace, rail transit and automotive. However, the disadvantages of effective damping area, unstable damping performance, easy fatigue, and aging, greatly limited the further application of rubber materials. Thus, it is important to develop novel modified rubber damping materials. Herein, polyamidoamine dendrimers with terminal-modified phenolic hydroxyl and amine groups (G2 PAMAM-H) were designed and used as modifiers to improve the damping performance of chlorinated butyl rubber (CIIR). The results showed that the modification of G2 PAMAM by diphenolic acid can avoid its aggregation in the CIIR matrix. CIIR/G2 PAMAM-H nanocomposites exhibited high tan *δ*_max_ of 1.52 and wide damping temperature region of 140°C (tan *δ *> 0.55)at a very low loading (4.32 wt.%), which were strongerthan that of pure CIIR and CIIR/G2 PAMAM nanocomposites. In addition, these nanocomposites also exhibited a unique self-healing ability by multiple hydrogen bonds, which can effectively extend the life of the rubber material in actual production. Therefore, the dendrimer modification provided unique development opportunities for elastomers in certain highly engineered fields, such as vehicles, rail transit, aerospace, etc.

## GRAPHICAL ABSTRACT

**Figure:**
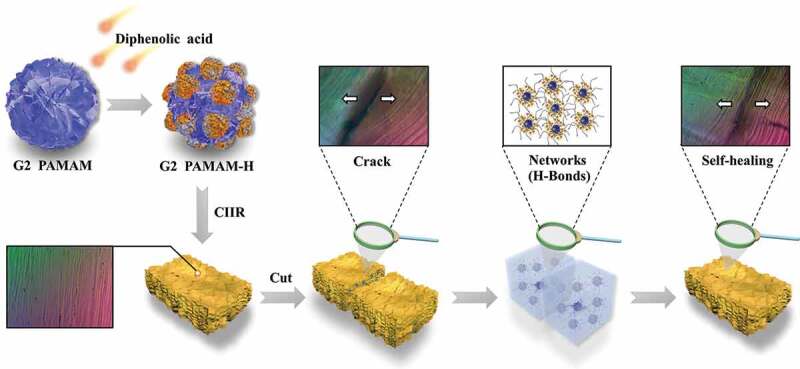

## 1. Introduction

Damping materials have a strong appeal in mechanical engineering applications due to their good vibration and noise reduction capabilities [1,2]. Especially in aerospace, rail transportation and other fields, the demand for high-performance damping materials was growing rapidly and continuously [3,4]. Rubber materials were one of the most commonly used damping materials because of their good mechanical properties and damping capacity [5]. However, there are somedisadvantagessuchasnarrow effective damping area, unstable damping performance, easy fatigue, and aging in practical use, limits their wide application [6,7]. Therefore, it is still important to develop better rubber shock-absorbing materials. The ideal rubber damping material should not only have good damping capacity, but also have good mechanical properties and durability [8,9].

Based on the principle of energy dissipation, several methods have been developed to improve rubber damping performance, for example, interfacial friction, the use of photoelectric conversion and the introduction of non-covalent bonds. The mechanism of interface damping is mainly attributed to the interfacial friction between the matrix molecular chains and the filler, and thereby improves the damping performance of polymer materials [10,11]. For example, Agrawal*et al* .[12] applied boron nitride nanotubes and carbon nanotubes to PLC (poly-lactic acid and poly-ε-caprolactone copolymer) to improve its damping performance. Interfacial friction between nanotubes and polymer matrix greatly improved the damping behavior of the composite materials. The principle of photoelectric conversion is to convert the kinetic energy of the molecular chains into other energy forms, to promote the dissipation of energy, and thereby improves the damping performance of polymer materials [13,14]. Zhang et al [15]. prepared a novel multi-walled CNTs/CT/F-PAEK-b-PDMS (carbon nanotube/barium titanate/polyaryl ether ketone-polydimethylsiloxane block copolymer) piezoelectric damping composite material. In detail, barium titanate converted part of the mechanical energy into electric energy, and multi-walled carbon nanotubes converted electric energy into heat energy, which realized energy dissipation and improved the damping performance of the composite material. However, this method is difficult to apply to the field of electrical insulation because it may improve the conductivity of the composites. The introduction of reversible sacrificial bonds to enhance the damping performance of polymer elastomers has become the focus of research in recent years [16]. In short, a large amount of energy can be effectively consumed by destroying these reversible sacrificial bonds in the system, thereby improving the toughness and damping properties of the materials [17,18]. For instance, Zhao*et al.* [19,20] reported methods of forming hydrogen bonds by adding small organic polar molecules (hindered phenols, hindered amines) to rubber systems. The dynamic energy dissipation of hydrogen bonds can significantly improve the damping property of the elastomers. However, small organic polar molecules tend to migrate to the surface of the elastomer, which makes the material properties unstable and limits the use of this method [21]. To solve this problem, one of the effective ways is to prepare large molecular weight polar molecules, reduce the mobility of the polar molecules and stabilize the internal structure of the composites.

In this study, we aim to improve the damping performance and aging resistance of rubber, using the abundant surface groups and special cavity structure of dendrimers. The terminal groups of the second-generation polyamidoamine (G2 PAMAM) were modified with phenolic hydroxyl groups to obtain a novel dendrimer (G2 PAMAM-H) with nineteen phenolic hydroxyl groups and six amine groups.G2 PAMAM-H was added as a modifier to the CIIR matrix to obtain novel CIIR nanocomposites. Compared with the original G2 PAMAM, the modification of G2 PAMAM by diphenolic acid can avoid its aggregation in the CIIR matrix, so that dendrimers are more uniformly dispersed in the CIIR matrix. First, the amine groups and the hydroxyl groups make the interface between G2 PAMAM-H and CIIR have a strong interaction, and the CIIR molecular chains can fill the cavity structure of G2 PAMAM-H. This makes G2 PAMAM-H and CIIR have good compatibility, and the internal structure remains relatively stable when the nanocomposites are stretched. Then, the dense phenolic hydroxyl and amine groups on the surface of the dendrimer can form multiple hydrogen bonds (including G2 PAMAM-Hintramolecular hydrogen bonds and intermolecular hydrogen bonds) in the system. These multiple hydrogen bonds have higher bond energy than single hydrogen bonds formed by small polar molecules. When an elastomeric material was subjected to cyclic stress, these multiple hydrogen bonds break to dissipate energy-and damping performance can be enhanced. Due to the reversibility of hydrogen bonding, unloading stress can restore the hydrogen bonding, thus forming continuous damping performance. In addition, multiple hydrogen bonds also endow the CIIR nanocomposites with a special self-healing ability, which provides a new solution for improving the self-healing property of traditional-vulcanized rubber.

## 2. Materials and methods

### 2.1. Materials

Ethylenediamine (98.5%), dimethyl sulfoxide(DMSO, 99.5%)and methanol (99.5%)were supplied by Shanghai Seaview Biochemical Technology Co. Ltd. (Shanghai, China), and methyl acrylate (97%) was purchased from Shanghai Lingfeng Chemical Reagent Co. Ltd. (Shanghai, China). Dimethyl sulfoxide (98%), diphenolic acid (97%),1-ethyl-3-(3ʹ-dimethylaminopropyl) carbodiimide (98%) and N-hydroxysuc-cinimide (98%) were purchased from Shanghai Macklin Biochemical Co. Ltd. (Shanghai, China). All of the above reagents were purified before use. Deuterium oxide (99.9 atom%D) and dimethyl sulfoxide-d6 (99.9 atom·%D) were purchased from Shanghai Saan Chemical Technology Co. Ltd. (Shanghai, China). Chlorinated butyl rubber(CIIR, Exxon 1066) was obtained from Exxon Mobil Chemical Industry Company (PA,USA). Rubber additives such as dibenzothiazole disulfide, magnesium oxide, tetramethyl thiuram disulfide, stearic acid, sulfur, and zinc oxide were obtained from the Market (Shanghai, China).

### 2.2. Preparation process of nanocomposites

#### 2.2.1. Preparation of G2 PAMAM

**Core**: Ethyl acrylate (20.64 g, 0.2400 mol) dissolved in methanol (40 mL) was cooled to 0°C using an ice bath under a nitrogen atmosphere. Ethylenediamine (EDA; 2.40 g, 0.3879 mol) dissolved in methanol (20 mL, 0.0399 mol) was dropwise added to the methyl acrylate solution through a constant pressure dropping funnel. The ice bath was kept for an additional 2 hours and then reacted at room temperature for 24 h. Excess methyl acrylate and methanol were removed using a rotary evaporator. The final product was obtained as colorless oil in a yield of 97.5% (15.77 g, 0.0390 mol).

**G0 PAMAM**: The core of PAMAM (1.00 g, 0.0025 mol) was dissolved in methanol (10 mL) under a nitrogen atmosphere, and was cooled to 0°C using an ice bath. Ethylenediamine (7.50 g, 0.1248 mol) was dissolved in methanol (20 mL) and dropwise added to a methanol solution of the core of PAMAM through a constant pressure dropping funnel under a nitrogen atmosphere. Then, it was reacted at room temperature for 4 days in an ice bath. Excess EDA, methyl acrylate, and methanol were removed using a rotary evaporator. Thereafter, a mixture of methanol/toluene (1/9) was subjected to azeotropic distillation until all EDA was removed by vacuum. Excess toluene was removed by azeotropic distillation with methanol. The final compound was obtained as colorless oil in quantitative yield (1.21 g, 0.0235 mol).

**G0.5 PAMAM, G1.5 PAMAM**: Methyl acrylate (3 equivalent (eq). per dendrimer amine surface group) was dissolved in methanol (usually in the same volume as methyl acrylate) and cooled to 0°C with an ice bath. It was added dropwise to a methanol solution(10 w/w %) of G0 PAMAM and G1.0 PAMAM (1 eq.) under a nitrogen atmosphere using a constant pressure dropping funnel. The ice bath was kept for an additional 2 hours and then reacted at room temperature for 2 days. The solvent and excess methyl acrylate were removed on a rotary evaporator. The product was dialyzed against methanol for 2 days using a dialysis membrane having molecular weight cut off (MWCO) of 500 and 1500, respectively. The methanol solvent was then removed on a rotary evaporator. A half-generation dendrimer in the form of pale yellow oil was obtained.

**G1 PAMAM, G2 PAMAM**: Half-generation PAMAM (1 eq.) was dissolved in methanol (10 w/w %) and cooled to 0°C using an ice bath. Ethylenediamine (25 eq. per ester surface group) was dissolved in methanol (25% of the ethylenediamine volume), and slowly added dropwise to a methanol solution of G0.5 PAMAM and G1.5 PAMAM under a nitrogen atmosphere using a constant pressure dropping funnel. The ice bath was kept for an additional 2 hours and then reacted at room temperature for 4 days. Post-treatment is accomplished by removing as much vacuum as possible to remove methanol and EDA. Azeotropic distillation was then carried out with a mixture of methanol/toluene (1/9) until all EDA was removed. Excess toluene was removed by azeotropic distillation with methanol. The product was dialyzed against methanol for 2 days using a dialysis membrane having molecular weight cut off (MWCO) of 1000 and 3000, respectively. The methanol solvent was then removed on a rotary evaporator. A whole generation of dendrimer in the form of pale yellow oil was obtained.

#### 2.2.2. Preparation of G2 PAMAM-H

The diphenolicacid (8.45 g, 0.03 mol) was first dissolved in DMSO (200 mL), and then 1-ethyl-3-(3ʹ-dimethylaminopropyl) carbodiimide (EDC) (57.11 g, 0.30 mol) and N-hydroxysuccinimide (NHS) (34.28 g, 0.30 mol) were added to a solution of diphenolicacid in DMSO. The mixture was added dropwise to a solution of G2 PAMAM (5.00 g, 1.54 mmol) in DMSO (15 mL). It was then reacted at room temperature for 3 days. The dialysis membrane having a molecular weight cut off (MWCO) of 3,500 was used. Then, the reaction mixture was dialyzed against DMSO (9 times, 2 L) for 2 d and dialyzed with water (18 times, 4 L) for 3 d. Water was removed from the freeze dryer to obtain G2 PAMAM-H as a pale yellow powder, and it was stored at −20°C for later use.

#### 2.2.3 Preparation of CIIR/G2 PAMAM-H nanocomposites

The CIIR rubber was masticated on the rubber mixer for 3 min, keeping the roller temperature below 45°C. Then, the calculated amount of vulcanization aid (stearic acid, magnesium oxide, accelerator dibenzothiazole disulfide, accelerator tetramethyl thiuram disulfide, sulfur and zinc oxide) were added and tampered twice after each addition. Finally, the G2 PAMAM-H powder was added to the rubber mixture to make it evenly dispersed in the rubber matrix. The temperature and pressure of the flat vulcanizer were set to 160°C and 10 MPa, and the uniform CIIR was kneaded, hot pressed and vulcanized for 30 min to prepare a sample for measurement. For comparison, pure CIIR and CIIR/G2 PAMAM nanocomposites were prepared in the same way. According to the above curing method, a CIIR blend was prepared according to the proportions of the samples listed in Table 1.

**Table 1.** Composition of CIIR/G2 PAMAM-H blends

Components	Parts per hundred of rubber (phr)
CIIR	100.00
G2 PAMAM	5/10
G2 PAMAM-H	0/1/3/5/10/15
Dibenzothiazole disulfide	2.00
Magnesium oxide	0.15
Tetramethyl thiuram disulfide	1.50
Stearic acid	1.00
Sulfur	1.00
Zinc oxide	5.00

### 2.3. Calculation method of inner cavity diameter of G2 PAMAM-H aggregates(SAXS)

In order to further verify the entanglement of G2 PAMAM-H with CIIR molecular chains, small-angle X-ray scattering (SAXS) spectroscopy was used on G2 PAMAM-H and CIIR/G2 PAMAM-H nanocomposites. For G2 PAMAM-H and nanocomposites, when the system conforms to Porod’s theorem, its scattering may show a certain value in the large wave vector region through the ln [*q*^3^I (*q*)]-*q*^2^ transformation [22].

(1) q=4πsinθ/λ

(2) $$\underset{q\to \infty }{\lim}\left\{\ln\left[q^{3}I\left(q\right)\right]\right\}=\ln K$$

Where *q* was the scattering vector, 2θ was the scattering angle, λ was the incident

X-ray wavelength, *I*(*q*) was the fuzzy scattering intensity (long slit collimation), and Kwas called the Porod constant. When the ln [*q*^3^I(*q*)]-*q*^2^ curves in the large wave vector region do not show a fixed value, but an upward trend. This may be due to the pore size of the G2 PAMAM-H [23]. The average pore diameter can be calculated by fitting the Guinier curve with the following formula:

(3) $$\ln I\left(q\right)=\ln I\left(0\right)-\frac{1}{3}{R_{g}}^{2}q^{2}$$

When the actual system obeys Guinier’s law, ln*I*(*q*)-*q*^2^was approximately linear in the low *q* region. The linear fit gives the slope of the straight line. *R*_g_ can be obtained from the slope of the straight line, and the characteristic length of the scatter can be converted. The formula was as follows:

(4) $$R_{g}={\left(3B\right)}^{0.5}$$

(5) $$R_{a}={\left(5/3\right)}^{0.5}R_{g}$$

R_a_ is the internal cavity radius of G2 PAMAM-H.

### 2.4. Characterization techniques

The morphology and microstructure of the G2 PAMAM and G2 PAMAM-H were investigated by transmission electron microscopy (TEM, FEI Tecnai G2 F20, USA)andatomic force microscopy (AFM, NanoManVS, Bruker, Germany). The structure and chemical composition of each generation of polyamidoamine dendrimers were characterized by Fourier-transform-infrared spectroscopy (FTIR, AVATAR 370, Nicolet, USA), ^1^H and ^13^C nuclear magnetic resonance (NMR) and 2D diffusion-ordered spectroscopy (DOSY, Bruker Avance-III 400 MHz, Switzerland) and matrix-assisted laser desorption/ionization time of flight mass spectrometry (MALDI-TOF-MS, Voyager DE-STR, USA; LC-MS, PR-LCMS-2020, Japan). The nanocomposite samples were cryo-embedded by Lycra uc7 equipment, and the morphology and microstructure of the nanocomposites sections were investigated by transmission electron microscope (TEM, Jeol 1230, Japan), atomic force microscopy (AFM, Bruker Dimension Icon, Germany) andscanning electron microscopy (SEM, Hitachi S4800, Japan). SAXS spectra were recorded using a SAXSess mc2 (Anton Paar, Austria) scatterometer with a 0.154 nm monochromatic radiation. The tensile tester (GT-TCS-2000, Gotech Testing Machines Co., Ltd., China) and dynamic thermomechanical analyzer (TA  USA, Q800) were applied to measure the mechanical and dynamic mechanical properties of the nanocomposites. The temperature-dependent FTIR spectra of nanocomposite sections were recorded on a Nicolet iS50 (USA) FTIR spectrometer combined with a heating device.

## 3. Results and discussion

### 3.1. Analysis of the synthesized G2 PAMAM and G2 PAMAM-H

**Figure 1.**
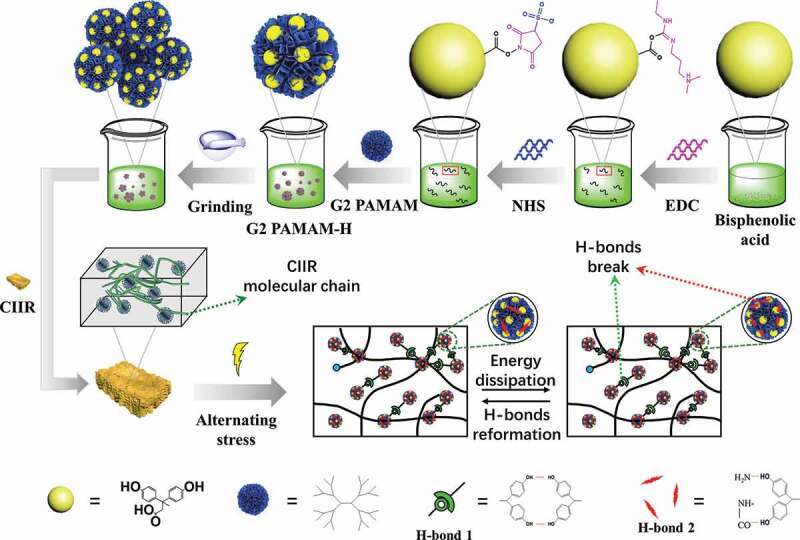
Schematic presentation of preparation process ofG2 PAMAM-H and CIIR/G2 PAMAM-H nanocomposites

**Figure 2.**
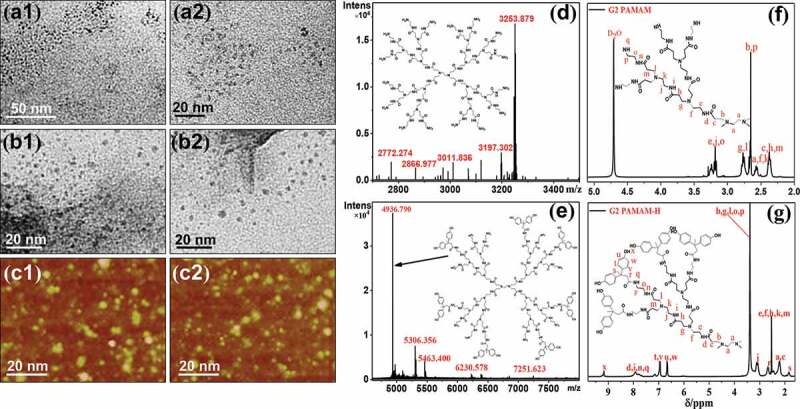
(a1,2) TEM images of G2 PAMAM, (b1,2) TEM images of G2 PAMAM-H, (c1,2) AFM images of G2 PAMAM-H, (d) LC-MS of G2 PAMAM, (e) MALDI-TOF-MS of G2 PAMAM-H, (f) ^1^H-NMR of G2 PAMAM, (g) ^1^H-NMR of G2 PAMAM-H

In this study, G2 PAMAM was obtained by the alternating reaction of ethylenediamine and methyl acrylate (Fig. S1).As shown in Figure 1, the amine-terminated of G2 PAMAM was modified by the diphenolic acid through EDC/NHS coupling reaction.FTIR and NMR spectra were used to identify the structure of G2 PAMAM and G2 PAMAM-H. The characteristic peaks at 3437 and 1070 cm^−1^ belonged to the stretching vibration absorption peaks of phenolic compounds -OH and -(C-O)- (Fig. S2). The characteristic peak at 9.1 ppm can be assigned to the hydroxyl of the phenolic hydroxyl groups on G2 PAMAM-H and the peaks at 6.78 and 6.96 ppm can be attributed to the benzene ring on the phenolic hydroxyl groups (Figure 2g). The characteristic peaks at 156.2, 138.9, 126.8 and 118.1 ppm on the ^13^C-NMR of G2 PAMAM-H belonged to the benzene ring on the phenolic hydroxyl groups (Fig. S3), confirming the phenolic hydroxyl groups were successfully grafted on the surface of G2 PAMAM. By calculating the integrated area of the ^1^H-NMR spectrum (Figure 2g), the number of phenolic hydroxyl groups attached to each G2 PAMAM was estimated as 19.3(Table S1). MALDI-TOF-MS test was performed on the synthesized G2 PAMAM-H to verify the integration results of ^1^H-NMR. Figure 2e shows that the molecular weight of G2 PAMAM-H was mainly distributed around 5,000. There were also small distributions at 5306, 5463, 6230, 7251, etc. The final synthesized G2 PAMAM-H had an average molecular weight, 5485, which was consistent with the integration result of ^1^H-NMR (Table S2).

TEM and AFM images of G2 PAMAM and G2 PAMAM-H are shown in Figure 2a and c(1,2), respectively. TEM images showed that G2 PAMAM was a spherical molecule with a diameter of about 3 nm (Figure 2a1,2). The diameter of G2 PAMAM-H modified by diphenolic acid was increased to 4 nm. G2 PAMAM-H molecules also exhibited a spherical structure in a three-dimensional space with uniform size (Figure 2b,c). Compared to G2 PAMAM, G2 PAMAM-H exhibited a larger internal cavity [24,25]. The increase of the cavity area inside the molecule facilitated the intertwining of the rubber molecular chains with the branches of G2 PAMAM-H [26].

### 3.2. Morphology observation of CIIR/G2 PAMAM-H nanocomposites

**Figure 3.**
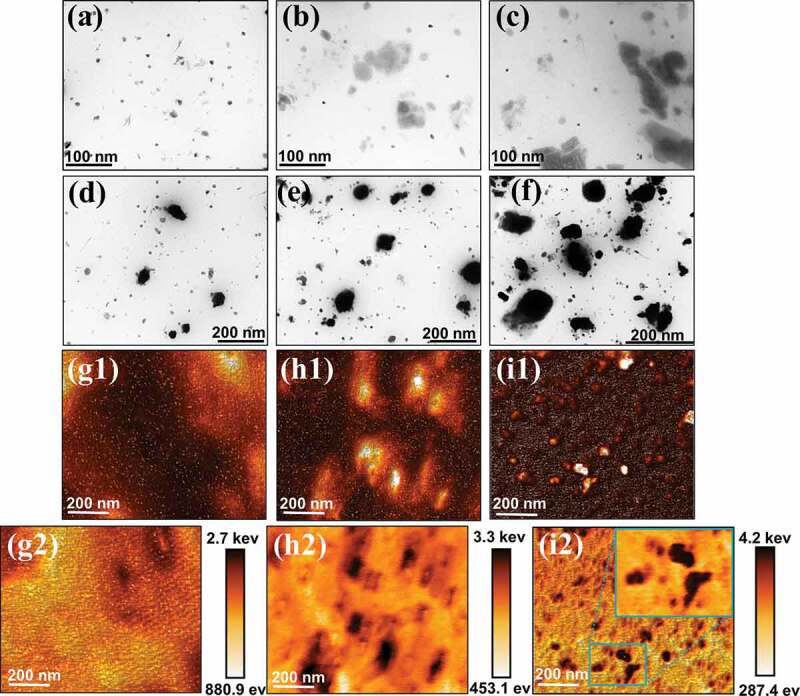
TEM images (a) pure CIIR, (b) CIIR/5phr G2 PAMAM, (C) CIIR/10phr G2 PAMAM, (d) CIIR/1phr G2 PAMAM-H, (e) CIIR/5phr G2 PAMAM-H, (f) CIIR/10phr G2 PAMAM-H; AFM images of (g1-g2) pure CIIR, (h1-h2) CIIR/10phr G2 PAMAM, (i1-i2) CIIR/5phr G2 PAMAM-H

The morphology of pure CIIR, CIIR/G2 PAMAM and CIIR/G2 PAMAM-H nanocomposites was observed by TEM and AFM. The pure CIIR morphology is shown in Figure 3a,g. The pure CIIR showed a relatively smooth crosssection, with dispersed particles(rubber additives) in a diameter of about 10 nmand no obvious particle agglomeration (Figure 3a). After adding G2 PAMAM, the TEM image showed that G2 PAMAM existed as agglomerated structure in the CIIR matrix, and the agglomerates size was about 100 nm. With the increase of G2 PAMAM content, the agglomerates size also increased (panels b and c). After adding 1and 5phr G2 PAMAM-H, it was observed that spherical agglomerates with uniform size and diameter of about 50 nm were evenly distributed in the CIIR (Figure 3d,e). As the content of G2 PAMAM-H increased to 10 phr, the TEM image showed that the size of the partially aggregated structure increased to 100 nm. This serious agglomeration was not conducive to enhancing mechanical properties. Although the added G2 PAMAM and G2 PAMAM-H showed agglomeration, the nanocomposites sheet were continuous and uniform in microscopic appearance without cracks (Figure 3b-f). Figure 3g-i present the AFM image of the cross section of the nanocomposites obtained by dissipative scanning in Quantitative Nano Mechanics (QNM) mode. Compared with pure CIIR (Figure 3g1,g2), CIIR/5 phr G2 PAMAM (Figure 3h1,h2) and CIIR/5 phr G2 PAMAM-H (Figure 3i1,i2) nanocomposites exhibited a clear sea-island phase structure. The marine phase represents CIIR, while the dark island phase corresponds to G2 PAMAM and G2 PAMAM-H particles.The color scale (from light to dark) corresponds to an increase in energy consumption capacity from low to high (Figure 3g2,h2,i2). This result demonstrates that the energy dissipation capability of the G2 PAMAM-H part was stronger than that of the G2 PAMAM and CIIR matrix. The agglomeration phenomenon of G2 PAMAM was more serious, which was not conducive to improving the energy consumption capacity of nanocomposites [27,28].The dissipation ability around G2 PAMAM and G2 PAMAM-H particles had a clear transition area (Figure 3b3), which was attributed to the following two reasons: (1) Multiple hydrogen bonds were generated by dendrimers in the system; (2) The cavity of dendrimers was filled with CIIR molecular chains, and the two phases were fused.

**Figure 4.**
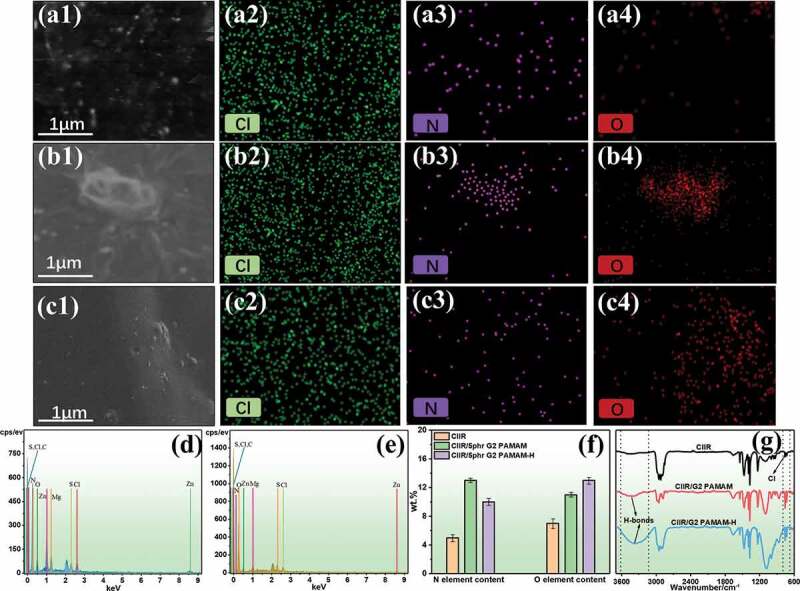
SEM-EDS mapping of(a1-a4) pure CIIR, (b1-b4) CIIR/5phr G2 PAMAM,(c1-c4) CIIR/5phr G2 PAMAM-H, Element content of (d) pure CIIR; (e) CIIR/5phr G2 PAMAM-H, (f) percentage of N and O in CIIR nanocomposites,(g) FTIR of pure CIIR, CIIR/5phr G2 PAMAM and CIIR/5phr G2 PAMAM-H

The internal morphology and composition of CIIR/G2 PAMAM-H nanocomposites were further studied by SEM and energy-dispersive spectroscopy (EDS). As shown in Figure 4a-c, elements Cl, N and O were detected, which provided specific distribution information of elements within the materials. After adding G2 PAMAM, the distribution of N and O elements became uneven (Figure 4b3,b4). This was because the G2 PAMAM molecular structure contained many N and O elements, and G2 PAMAM tended to form large agglomerate. It was observed that the O elements of the nanocomposites were not uniformly distributed after addition of G2 PAMAM-H (Figure 4c3,c4). The uniform distribution of nitrogen was due to the reduction of the N content of the G2 PAMAM-H obtained after diphenolic acid replaced the end groups of G2 PAMAM. In addition, there was also a small amount of N element in the rubber additives (dibenzothiazole disulfide, tetramethyl thiuram disulfide), resulting in inconsistent distribution of nitrogen and oxygen. The large number of amides and hydroxyl groups contained in G2 PAMAM-H may lead to an increase in the content of O elements in the nanocomposites (Figure 4f). However, there was no significant difference in the distribution of the Cl element between pure CIIR and nanocomposites (Figure 4a2,b2,c2), indicating that the addition of G2 PAMAM andG2 PAMAM-H did not affect the uniformity of the system. The inner cavity of dendrimers was entangled with the molecular chains of rubber, which cannot destroy the integrity of CIIR. Comparing the infrared spectra of pure CIIR, CIIR/5 phr G2 PAMAM and CIIR/5 phr G2 PAMAM-H (Figure 4g), there was no obvious change in the Cl peak, which proved that the Cl in CIIR cannot form a covalent bond with the surface amino group in the dendrimer. The infrared spectra of CIIR/5 phr G2 PAMAM and CIIR/5 phr G2 PAMAM-H exhibited hydrogen bond peaks at 3000–3500 cm^−1^. Moreover, the hydrogen bond peaks formed by CIIR/5 phr G2 PAMAM-H were stronger than CIIR/5 phr G2 PAMAM, indicating that there were more hydrogen bonds formed in the CIIR/5 phr G2 PAMAM-H system.

### 3.3. Tensile properties of CIIR/G2 PAMAM-H nanocomposites

**Figure 5.**
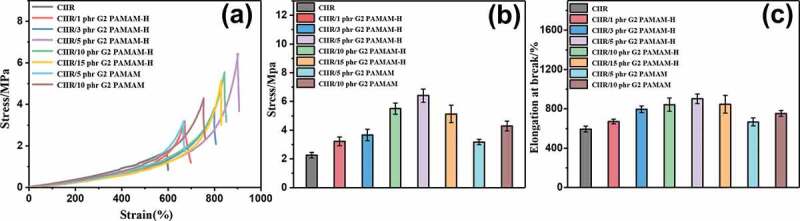
(a) stress-strain curves of CIIR nanocomposites, (b) tensile strength and (c)elongation at break of CIIR nanocomposites

The mechanical properties of CIIR with different ratios of G2 PAMAM and G2 PAMAM-H were characterized by static uniaxial tensile tests (Figure 5). The addition of G2 PAMAM and G2 PAMAM-H enhances the mechanical properties of nanocomposites. The mechanical properties of CIIR/G2 PAMAM-H nanocomposites are significantly higher than those of CIIR/G2 PAMAM nanocomposites containing the same fraction, which proves that the modification of diphenolicacid improves the enhancement effect of dendrimers. When the fraction of G2 PAMAM-H added is 1–5 phr, as the fraction of G2 PAMAM-H increased, the maximum fracture strain and tensile fracture length of CIIR nanocomposites increased significantly (Figure 5b,c). By comparing pure CIIR and CIIR/5 phr G2 PAMAM-H, the tensile strength and elongation at break were increased from 1.95 MPa and 410% to 6.45 MPa and 920%, respectively. It was deduced that G2 PAMAM-H was added as a modifier into the CIIR matrix.G2 PAMAM-H has a cavity structure inside. These cavity structures can be penetrated by rubber molecular chains to form physical cross-linking points.Under the tensile stress, the friction between G2 PAMAM-H and CIIR molecular chains consumed part of the energy, which increased the toughness and tensile strength of these CIIR nanocomposites [29,30]. In addition, lots of polar groups were distributed on the surface of G2 PAMAM-H molecules. A large number of hydrogen bonds can be generated with the rubber molecular chains in the system [31–33]. Suckow*et al* [34]. used carbonyl and imine groups to form hydrogen bonds in rubber systems, which also enhanced the mechanical properties of nanocomposites. These sacrificial hydrogen bonds possessed energy dissipation capabilities when they were stretched. A portion of the energy was absorbed by the broken hydrogen bonds, resulting in a corresponding increase in strain at break and toughness. When the addition amount of G2 PAMAM-H was 10phr and 15phr, the mechanical properties of the nanocomposites have worsened. This was because the polar groups on the surface of G2 PAMAM-H tended to agglomerate. As the content of G2 PAMAM-H increased, the agglomeration phenomenon became more serious, which may worsen the mechanical properties of nanocomposites.

### 3.4. Dynamic mechanical properties of CIIR/G2 PAMAM-H nanocomposites

**Figure 6.**
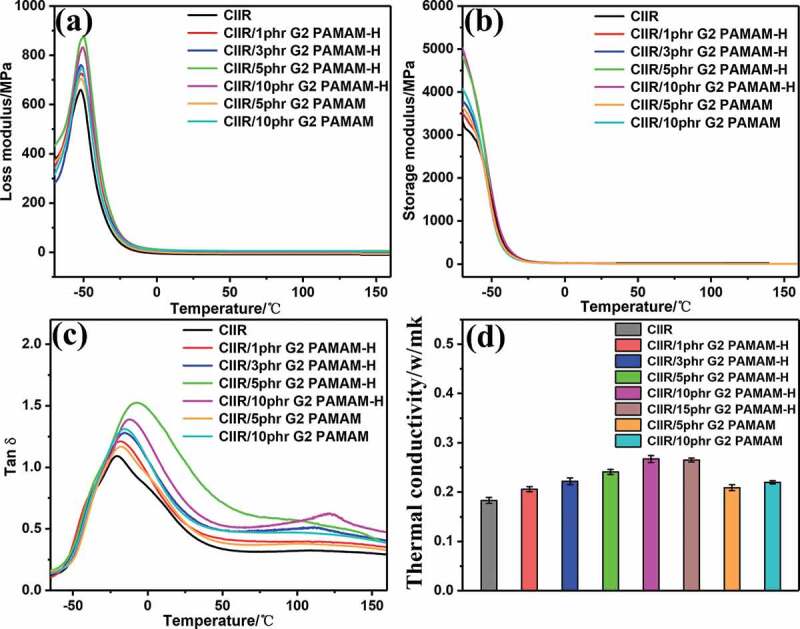
(a) loss modulus E’’, (b) elastic modulus E’, (c) damping factor tan *δ* of CIIR/G2 PAMAM-H nanocomposites, (d) thermal conductivity and thermal resistance coefficients of CIIR/G2 PAMAM-H nanocomposites

Under the action of alternating stress, loss tangent (tan *δ*) and modulus (E’, E’’) with temperature of pure CIIR and CIIR/G2 PAMAM-H nanocomposites are shown in Figure 6. Figure 6a,b present the loss modulus and storage modulus of nanocomposites as a function of temperatures. When the temperature was lower than T_g_, the storage modulus E’of the nanocomposites was higher than that of pure CIIR (Figure 6b). The hydrogen bonds formed by G2 PAMAM-H increased the storage modulus of the nanocomposites, and thus T_g_ moved toward the high temperatures. After reaching T_g_, the loss modulus E’’ of nanocomposites was increased (Figure 6a). The modulus was higher than the pure CIIR because the rubber molecular segment began to move. At this time, the hydrogen bonds alternately broke and recovered, consumed a certain amount of energy, and thus the loss modulus was increased. Tan *δ* (also known as damping factor) was the ratio of the loss modulus of the rubber to the storage modulus in each deformation cycle.

The higher the value of tan *δ*, the wider the corresponding temperature ranges were. This illustrated that the damping properties of the nanocomposites were significantly improved after the addition of G2 PAMAM and G2 PAMAM-H.The value of tan *δ* of CIIR/G2 PAMAM-H nanocomposites were significantly higher than those of CIIR/G2 PAMAM nanocomposites containing the same fraction. After the modification process, the number of polar groups on the surface of the dendrimer increased, and the number of hydrogen bonds formed in the composite system increased. In addition, the strength of hydrogen bonds formed by phenolic hydroxyl groups was higher than that of amine groups. resulting in the enhancement effect of G2 PAMAM-H better than G2 PAMAM. The tan *δ* of CIIR/5 phr G2 PAMAM-H was increased to 1.52. The tan *δ*_max_ was increased by 41% and 31% compared to the maximum value of the pure CIIR, 1.08 and CIIR/5 phr G2 PAMAM, 1.16. Compared to the temperature range (tan *δ *> 0.55), the temperature range of pure CIIR was 64°C (−45 ~ 19°C) and CIIR/5 phr G2 PAMAM-H owned an increased temperature range. Its value reached 140°C (−43 ~ 97°C), which was increased by 119% and102% compared to pure CIIR (64°C, −44 ~ 20°C) and CIIR/5 phr G2 PAMAM (70°C, −44 ~ 26°C). In addition, the effective damping area was moved toward high temperatures, which was advantageous for practical use. The enhancement of CIIR damping performance was related to the special structure of G2 PAMAM-H. As the temperature rose above T_g_, these nanocomposites were under the action of alternating stress. The rubber molecular chains and segments began to move. Physical friction was generated with addition of G2 PAMAM-H. In this process, kinetic energy was converted into heat and consumed a portion of the energy. In addition, the surface of G2 PAMAM-H owned a large number of polar groups. Many hydrogen bonds were formed in the system. These hydrogen bonds were alternately destroyed and reconstructed under the influence of external forces, and they also consumed part of the energy. The regenerability of the hydrogen bonds allowed the rubber to own sustained damping effect [35,36].

The practical application of damping materials was to convert external kinetic energy into thermal energy. Therefore, thermal conductivity was an important parameter for rubber in practical damping applications. The thermal conductivity of pure CIIR was 0.179 W m^−1^ K^−1^. With the addition of G2 PAMAM and G2 PAMAM-H, the thermal conductivity of the nanocomposites gradually improved (Figure 6d). The thermal conductivity of CIIR/10 phr G2 PAMAM-H was 0.251 W m^−1^ K^−1^. Compared with pure CIIR and CIIR/10 phr G2 PAMAM, the thermal conductivity was increased by 40% and23%, respectively. The improved thermal conductivity of the nanocomposites was due to the excellent thermal conductivity of G2 PAMAM-H itself. In addition, the added G2 PAMAM-H formed a large number of hydrogen bonds in CIIR. The cross-linking density of the system was increased (FigureS6), and thus the heat transfer was promoted [37].

### 3.5. Self-healing performance of CIIR/G2 PAMAM-H nanocomposites

**Figure 7.**
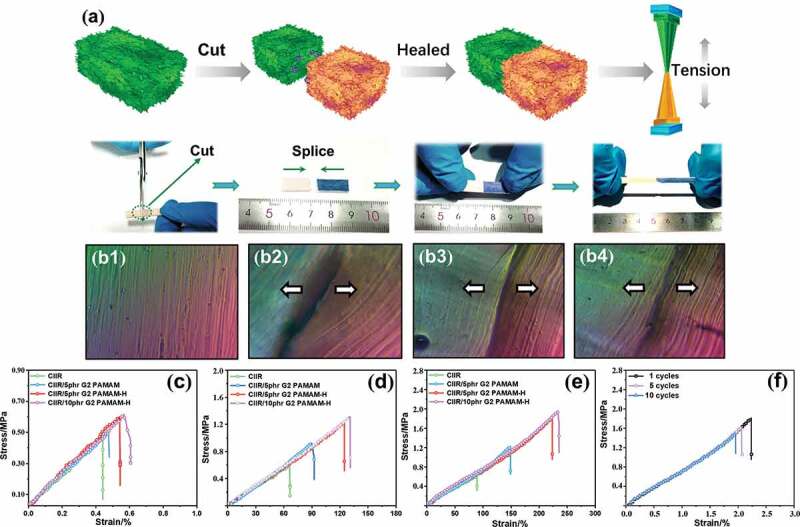
(a) schematic diagram of self-healing performance test method, surface morphology of CIIR/5phr G2 PAMAM-H nanocomposites (b1) intact section, (b2) cross-section after cutting, (b3) fracture surface after self-repairing for 5 min, (b4) fracture surface after self-repairing for 20 min. Stress-strain curves of self-repaired CIIR nanocomposites (c) self-healing time was 0.5 h, (d) self-healing time was 6 h, (e) self-healing time was 24 h, (f) stress-strain curves of CIIR nanocomposites with different cycles (one cycle: Self-healing 24 h after cutting)

CIIR/G2 PAMAM-H nanocomposites also exhibited some interesting self-healing behaviors. Figure 7b1 shows the complete rubber section. A clear crack on the fracture surface of the rubber nanocomposites was exhibited (Figure 7b2). The self-healing of the fracture surface at room temperature after 5 minis illustrated in Figure 7b3. Figure 7b4 shows the self-healing effect at room temperature for 20 min. It can be seen that the crack was significantly reduced and the fracture became smooth. In addition, the separated surface was well healed, almost no cracks were visible, and only some wrinkles were exhibited at the break.To verify this behavior, we performed a tensile test on the self-healed nanocomposites after shearing. As the recovery time was increased, the healing effect was increased significantly (Figure 7c-e). After recovery for 24 h,the CIIR/5phrG2 PAMAM-H nanocomposites can withstand 1.94 MPa stress. Compared to pure CIIR samples (0.61 MPa stress) and CIIR/5phrG2 PAMAM (1.21 MPa stress), they were increased by 220% and 61%, respectively (Figure 7e). In addition, as observed in Figure 7d,e, the addition of G2 PAMAM-H may also increase the self-healing ability of the nanocomposites. In the self-healing cycle test, after 10 cycles (one cycle: self-healing 24 h after cutting), CIIR/5phrG2 PAMAM-H still owned 196% strain and 1.69 MPa stress. Compared with the stress and strain after one cycle, the decrease was only 13%. The cyclic testfurther verified the self-healing stability of CIIR/5phr G2 PAMAM-H nanocomposites (Figure 7f).The reason for this increased self-healing ability was clearly related to G2 PAMAM-H. The dendrimers were special carriers of polar groups. The reversible multiple hydrogen bonds formed enhanced interactions between the two cutting and separated interfaces. The cavity structure inside G2 PAMAM-H allows it to be used as a physical cross-linking of the rubber molecular chain. Point, when the dendrimers interact with each other, G2 PAMAM and G2 PAMAM-H can drive the rubber molecular chain to move, and endowed CIIR with unique self-healing properties [38,39].

### 3.6. Toughening, damping, and self-healing mechanisms of CIIR/G2 PAMAM-H nanocomposites

The cavity of G2 PAMAM-H was assumed to be spherical. The pore diameter of G2 PAMAM-H was determined from the slope of the fitted curve to be about 19 nm, and the calculation result in pure CIIR was 10 nm. Combining the TEM and AFM images for the cross-section of the nanocomposites, it was speculated that this was the size of some additives added to CIIR. The calculated result of nanocomposites was 16 nm, which was very close to the pore size result of G2 PAMAM-H. We believed that the reason for the difference was that part of the pore size of G2 PAMAM-H was covered by CIIR molecular chains. This also proved that our proposed G2 PAMAM-Hcan serve as a physical crosslinking point in the CIIR system [40,41]. The cavity structure of G2 PAMAM-H can be filled, which demonstrated the good compatibility of G2 PAMAM-H in CIIR. The locally formed structure was similar to interpenetrating polymer network(IPN), and this may endow the nanocomposites with better damping performance when encountered alternating stress. This also made the nanocomposites more stable when performed in actual use.

**Figure 8.**
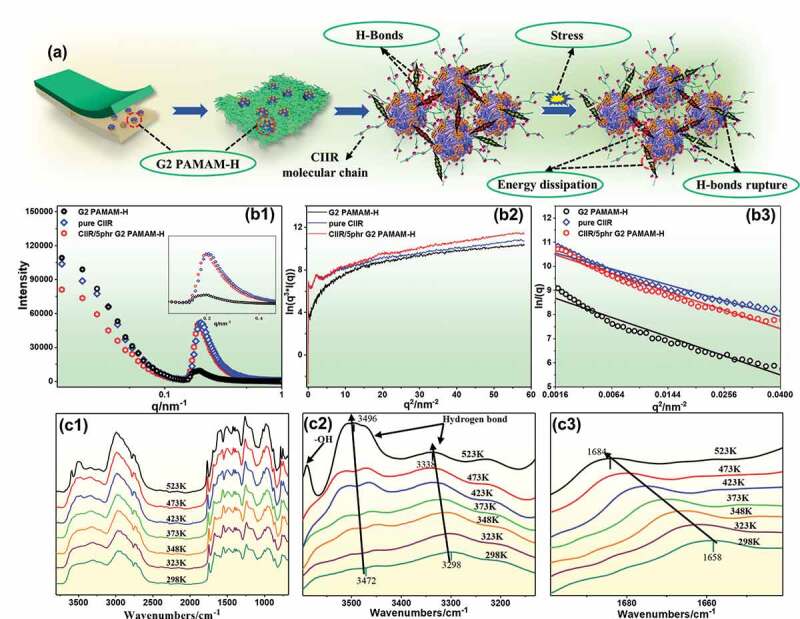
(a) schematic diagram of hydrogen bond energy dissipation generated by G2 PAMAM-H in CIIR matrix, (b) SAXS of G2 PAMAM-H, pure CIIR and CIIR/G2 PAMAM-H nanocomposites, (c1) In situ variable temperature infrared spectra of CIIR/5phr G2 PAMAM-H, (c2) blue shift of -OH, -NH_2_, (c3) blueshift of -C = O

The damping and self-healing mechanism of G2 PAMAM-H in CIIR nanocomposites was investigated by in situ variable temperature infrared spectroscopy. G2 PAMAM-H can theoretically form hydrogen bonds structure with -NH_2_, -OH, -CONH-, and these hydrogen bonds together with the characteristic peak positions of these groups may undergo changes with the increase of the temperature. Figure 8c2 presents an infrared absorption peak of a phenolic hydroxyl group from 3350 to 3600 cm^−1^. At 298 K, the maximum infrared absorption peak of the hydroxyl group was 3470 cm^−1^. As the temperature increased, its infrared absorption peak was increased and moved toward a higher wavenumber. Figure 8c2 also showed the stretching vibration absorption peak of the amino group (-NH_2_) in the amide bond. Between 3200 and 3350 cm^−1^, as the temperature increased, the absorption peak of the amino groups shifted to a higher wavenumber. Figure 8c3 shows the stretching vibration absorption peak of -C = O- in the amide bond. The peak intensity was confined between 1600 and 1700 cm^−1^. As the temperature increased, the peak shifted to a higher wavenumber. This blue shift phenomenon occurred for the following reasons: (1) The hydrogen bonds formed by G2 PAMAM-H in CIIR were thermally expanded during heating, and thus the hydrogen bonds became longer [42,43]. (2) During the heating process, the hydrogen bonds were broken and the energy of these special functional groups became high, and thus the structure was unstable and blue shift occurred. In addition, when the temperature was increased, the absorption peak of the phenolic hydroxyl groups split into two peaks (Figure 8c2), and a spike appeared at a higher frequency. This was caused by the decomposition of hydrogen bonds in the system. The hydrogen bonds were decomposed to produce free -OH radicals, which led to different chemical environments of the hydroxyl groups. Thus, the infrared absorption peak shifted and two different absorption peaks appeared. This also proved our previous inference about the rupture of hydrogen bonds.

## 4. Conclusions

In this research, G2 PAMAM-H was obtained by changing the surface groups of the G2 PAMAM with diphenolicacid. Then, CIIR nanocomposites with high mechanical strength, damping, and self-healing properties were obtained based on this novel nanoparticle. The morphology of G2 PAMAM and G2 PAMAM-H was characterized by TEM and AFM. The average diameter of the molecules was 3 nm and 4 nm, respectively. The dispersion of G2 PAMAM and G2 PAMAM-H nanoparticles in CIIR matrix were investigated by SEM-EDS, AFM and TEM. The results show that the modification of G2 PAMAM by diphenolic acid can avoid its aggregation in CIIR matrix. It shows that the G2 PAMAM-H is dispersed in the CIIR matrix in the form of agglomerates with a size of 50–100 nm. The cavity inside the G2 PAMAM-H was proved by SAXS to make it play a role in physical crosslinking.

In addition, the large amount of polar groups on the surface of G2 PAMAM-H made it form a large number of hydrogen bonds in the CIIR system, which enhanced the mechanical strength of CIIR and endowed it with a certain self-healing ability. The tan *δ*_max_ and damping temperature range (tan *δ *> 0.55) of CIIR nanocomposites expanded from 1.08 and 64°C to 1.52 and 140°C, respectively. The tensile strength and elongation at break were increased from 1.95 MPa and 410% to 6.45 MPa and 920%, respectively. The toughening, damping and self-healing mechanisms of hydrogen bonds in these nanocomposites were also verified by in-situ variable temperature infrared spectroscopy. Therefore, CIIR/G2 PAMAM-H nanocomposites with excellent mechanical properties and novel self-repairing properties have great potential in the fields of vehicles, rail transit, aerospace, and so on.

## Supplementary Material

Supplemental Material
