# Supplementary material for: Diphenolic acid-modified PAMAM/chlorinated butyl rubber nanocomposites with superior mechanical, damping, and self-healing properties
Source: Sci Technol Adv Mater. 2021 Jan 22;22(1):14–25. doi: 10.1080/14686996.2020.1861912 (PMC7832595; doi:10.1080/14686996.2020.1861912)
Supplement: Supplemental Material [file TSTA_A_1861912_SM9253.doc]

**Modified PAMAM/Chlorinated Butyl Rubber Nanocomposites with High Mechanical, Damping and Self-healing Properties**

Yao Lu^a^, Jincheng Wang^a^, *, Le Wang^a^, *, Shiqiang Song^a^

^a^College of Chemistry and Chemical Engineering, Shanghai University of Engineering Science, Shanghai, 201620, P.R.China

* Corresponding Author E-mail: [wjc406@126.com](mailto:wjc406@126.com)(Prof. Jincheng Wang), [wangle316@sues.edu.cn](mailto:wangle316@sues.edu.cn) (Dr. Le Wang)


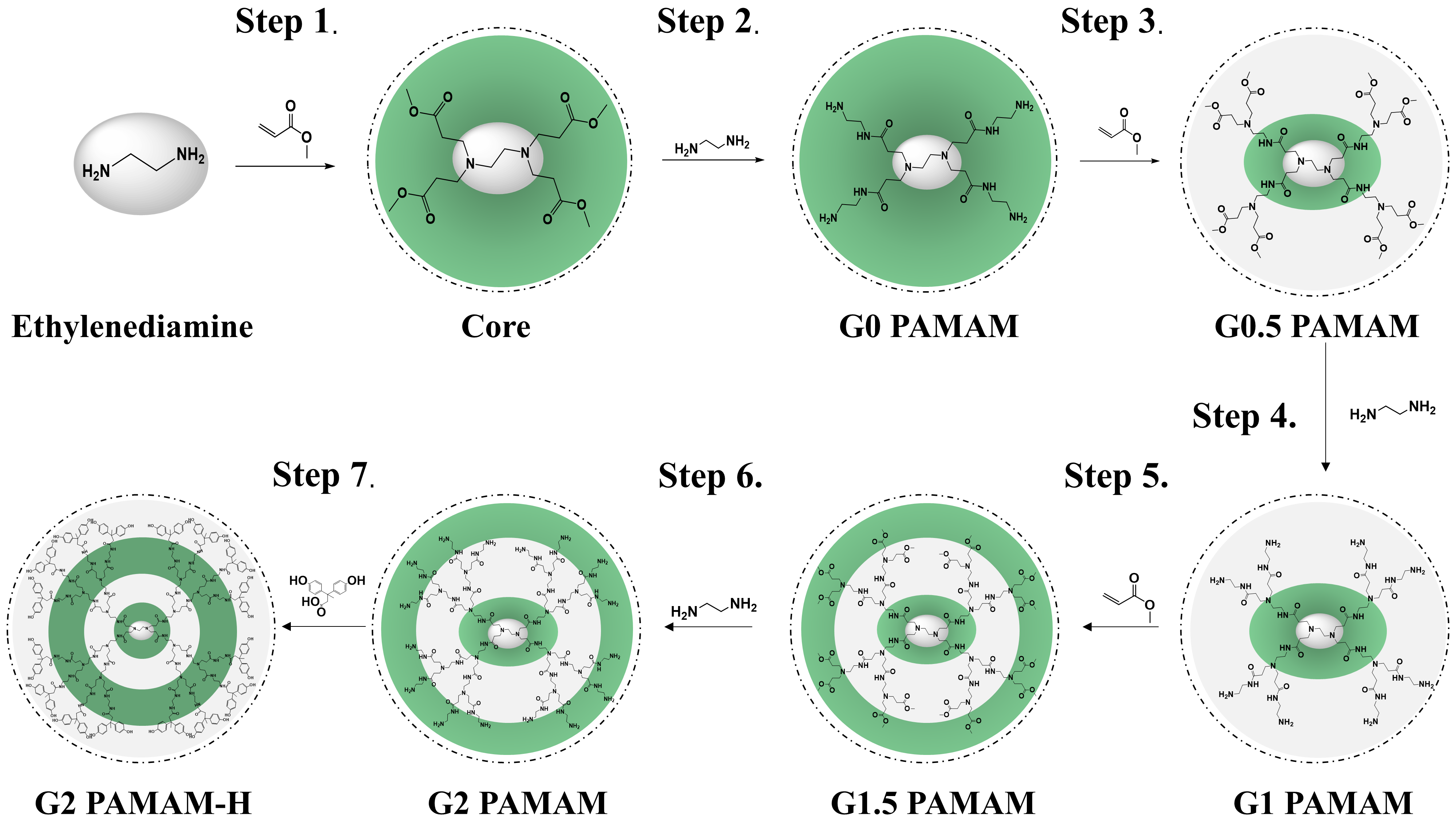


**Fig.S1** Preparation path of G2 PAMAM-H

**Fig.S2** FT-IR of Core, G1 PAMAM, G1.5 PAMAM, G2 PAMAM and G2 PAMAM-H


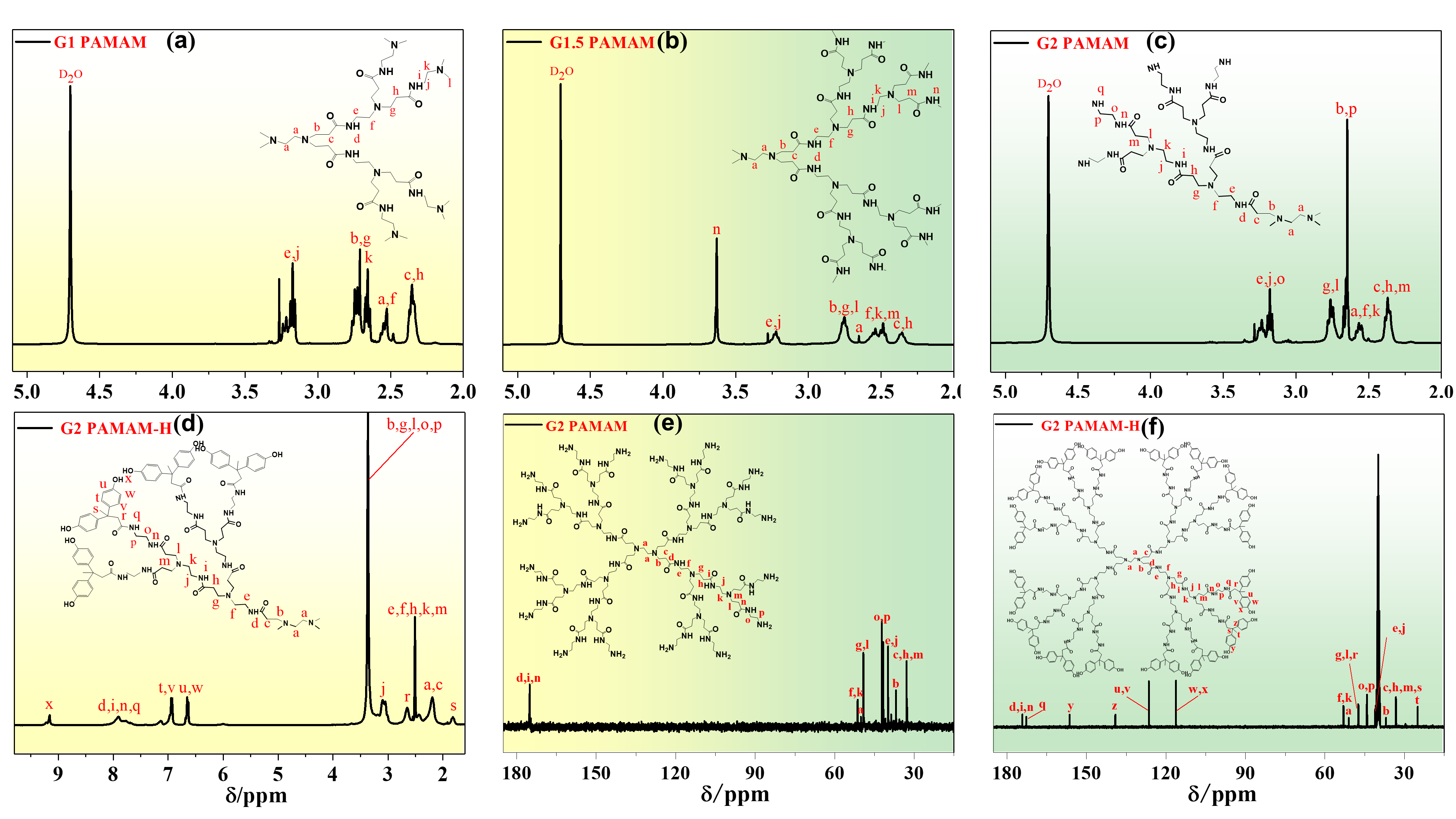


**Fig.S3** ^1^H-NMR and ^13^C-NMR partitioning of each generation of PAMAM dendrimer measured in deuterated water and deuterated dimethyl sulfoxide.

**Core:** Ethyl acrylate (20.64 g, 0.2400 mol) dissolved in methanol (40 mL) was cooled to 0 °C using an ice bath under a nitrogen atmosphere. Ethylenediamine (2.40 g) dissolved in methanol (20 mL, 0.0399 mol) was dropwise added to the methyl acrylate solution through a constant pressure dropping funnel. The ice bath was kept for an additional 2 hours and then reacted at room temperature for 24 hours. Excess methyl acrylate and methanol were removed using a rotary evaporator. The final product was obtained as colorless oil in a yield of 97.5% (15.77 g, 0.0390 mol).^1^H-NMR (400 MHz, D_2_O): δ = 2.35–2.41 (m, 8H), δ = 2.46 (t, 8H, ^3^*J* = 7.07 Hz), δ = 2.7 (t, 8H, ^3^*J* = 7.61 Hz), δ = 3.59 (s, 12H) ppm.

**G0 PAMAM:** The core of PAMAM (1.00 g, 0.0025 mol) was dissolved in methanol (10 mL) under a nitrogen atmosphere, and was cooled to 0 °C using an ice bath. Ethylenediamine (EDA; 7.50 g, 0.1248 mol) was dissolved in methanol (20 mL) and dropwise added to a methanol solution of the core of PAMAM through a constant pressure dropping funnel under a nitrogen atmosphere. Then, it was reacted at room temperature for 4 days in an ice bath. Excess EDA, methyl acrylate and methanol were removed using a rotary evaporator. Thereafter, a mixture of methanol/toluene (1/9) was subjected to azeotropic distillation until all EDA was removed by vacuum. Excess toluene was removed by azeotropic distillation with methanol. The final compound was obtained as a colorless oil in quantitative yield (1.21 g, 0.0235 mol).^1^H-NMR (400 MHz, D_2_O): δ = 2.37 (t, 8H, ^3^*J* = 7.05 Hz), δ = 2.51–2.54 (m, 8H), δ = 2.63 (t, 8H, ^3^*J* = 6.21 Hz), δ = 2.72 (t, 8H, ^3^*J* = 7.22 Hz), δ = 3.15 (t, 8H, ^3^*J* = 6.32Hz) ppm.

**G0.5, G1.5:** Methyl acrylate was dissolved in methanol (usually in the same volume as methyl acrylate) and cooled to 0 °C with an ice bath. It was added dropwise to a methanol solution of G0 and G1 under a nitrogen atmosphere using a constant pressure dropping funnel. The ice bath was kept for an additional 2 hours and then reacted at room temperature for 2 days. The solvent and excess methyl acrylate were removed on a rotary evaporator. The product was dialyzed against methanol for 2 days using a dialysis membrane having molecular weight cut off (MWCO) of 500 and 1500, respectively. The methanol solvent was then removed on a rotary evaporator. A half-generation dendrimer in the form of pale yellow oil was obtained. **G0.5 PAMAM:**^1^H-NMR (400 MHz, D_2_O): δ = 2.40 (t, 8H, ^3^*J* =7.08 Hz), δ = 2.49 (t, 16H, ^3^*J* = 7.16 Hz), δ = 2.51–2.53 (m, 8H), δ = 2.55 (t, 8H, ^3^*J* = 6.27 Hz), δ = 2.76 (t, 24H, ^3^*J* = 6.52 Hz), δ = 3.23 (t, 8H, ^3^*J* = 7.11 Hz), δ = 3.64 (s, 24H) ppm. **G1.5 PAMAM:**^1^H-NMR (400 MHz, D_2_O): δ = 2.36 (t, 24H, ^3^*J* = 7.08 Hz), δ = 2.49 (t, 32H, ^3^*J* = 7.08 Hz), δ = 2.49–2.53 (m, 4H),δ = 2.50–2.52 (m, 8H),δ = 2.54 (t, 16H, ^3^*J* = 6.23 Hz), δ = 2.64–2.66 (m, 8H), δ = 2.75 (t, 24H, ^3^*J* = 6.24 Hz), δ = 3.22(t, 24H, ^3^*J* = 6.39 Hz), δ = 3.69 (s, 48H) ppm.

**G1, G2:** Half-generation PAMAM was dissolved in methanol and cooled to 0 °C using an ice bath. Ethylenediamine was dissolved in methanol, and slowly added dropwise to a methanol solution of G0.5 and G1.5 under a nitrogen atmosphere using a constant pressure dropping funnel. The ice bath was kept for an additional 2 hours and then reacted at room temperature for 4 days. Post-treatment was accomplished by removing as much vacuum as possible to remove methanol and EDA. Azeotropic distillation was then carried out with a mixture of methanol/toluene (1/9) until all EDA was removed. Excess toluene was removed by azeotropic distillation with methanol. The product was dialyzed against methanol for 2 days using a dialysis membrane having molecular weight cut off (MWCO) of 1000 and 3000, respectively. The methanol solvent was then removed on a rotary evaporator. A whole generation of dendrimer in the form of a pale yellow oil was obtained. **G1 PAMAM:**^1^H-NMR (400 MHz, D_2_O): δ = 2.35 (t, 24H, ^3^*J* = 7.17 Hz), δ = 2.47–2.49 (m, 4H), δ = 2.55(t, 8H, ^3^*J* = 6.95 Hz), δ = 2.66 (t, 8H, ^3^*J* = 6.32 Hz), δ = 2.75 (t, 16H, ^3^*J* = 6.96 Hz), δ = 3.17 (t, 24H, ^3^*J* = 6.35 Hz) ppm. **G2 PAMAM:** ^1^H NMR (400 MHz, D_2_O): δ = 2.37 (t, 56H, ^3^*J* = 6.91 Hz), δ = 2.49–2.50 (m, 4H), δ = 2.56 (t, 24H, ^3^*J* = 7.10 Hz), δ = 2.63 (t, 32H, ^3^*J* = 6.3 Hz), δ = 2.66 (t, 56H, ^3^*J* = 6.79 Hz), δ = 2.76 (t, 56H, ^3^*J* = 7.20 Hz), δ = 3.15 (t, 56H, ^3^*J* = 6.58 Hz) ppm.^13^C-NMR (125 MHz, D_2_O): δ = 33.64, 37.05, 39.90, 41.50, 42.08, 49.28, 51.39, 174.65, 175.13 ppm.

**G2 PAMAM-H:**^1^H-NMR (400 MHz, DMSO-d6): δ = 1.70(s, 12H), δ = 2.19(m, 8H), δ = 2.43(m, 4H), δ = 2.51(m, 40H), δ = 2.65(m, 8H), δ = 3.08(m, 8H), δ = 3.37(m, 40H), δ = 6.65(d, 16H), δ = 6.94(d, 16H), δ =7.92(s, 16H) ,δ = 9.17(s, 16H)ppm.^13^C-NMR (125 MHz, D_2_O): δ=25.76,33.87, 38.35,41.57, 43.68, 48.37, 51.02,51.89, 118.12, 126.83, 138.91,156.25,172.75, 174.96 ppm.

The ideal number of grafted phenolic hydroxyl groups was 32, and the expected molecular weight was 7319. The actual number of grafted phenolic hydroxyl groups was inferred by ^1^H-NMR, LC-MS and MALDI-TOF-MS in the table below.

**Table S1.** Inferring the graft number of phenolic hydroxyl groups from the integrated area of ^1^H-NMR spectrum

|  | **x** | **r** | **e,f,h,k,m** |
| --- | --- | --- | --- |
| Peak area | 1 | 1.03 | 4.17 |
| Mean number of  phenolic hydroxyl groups per dendrimer | 19.3 | | |

**x, r, e, f, h, k, m** correspond to the marked positions in Fig S3(d)

**Table S2.** The inferred number of phenolic hydroxyl grafts from the molecular mass

|  | **G2 PAMAM** | **G2 PAMAM-H** |
| --- | --- | --- |
| Mw^a^ | 3106 | 5595 |
| Mean number of  phenolic hydroxyl groups per dendrimer | 18.4 | |

**^a^** Molecular weights were estimated by LC-MS (G2 PAMAM) and MALDI-TOF-MS (G2 PAMAM-H)


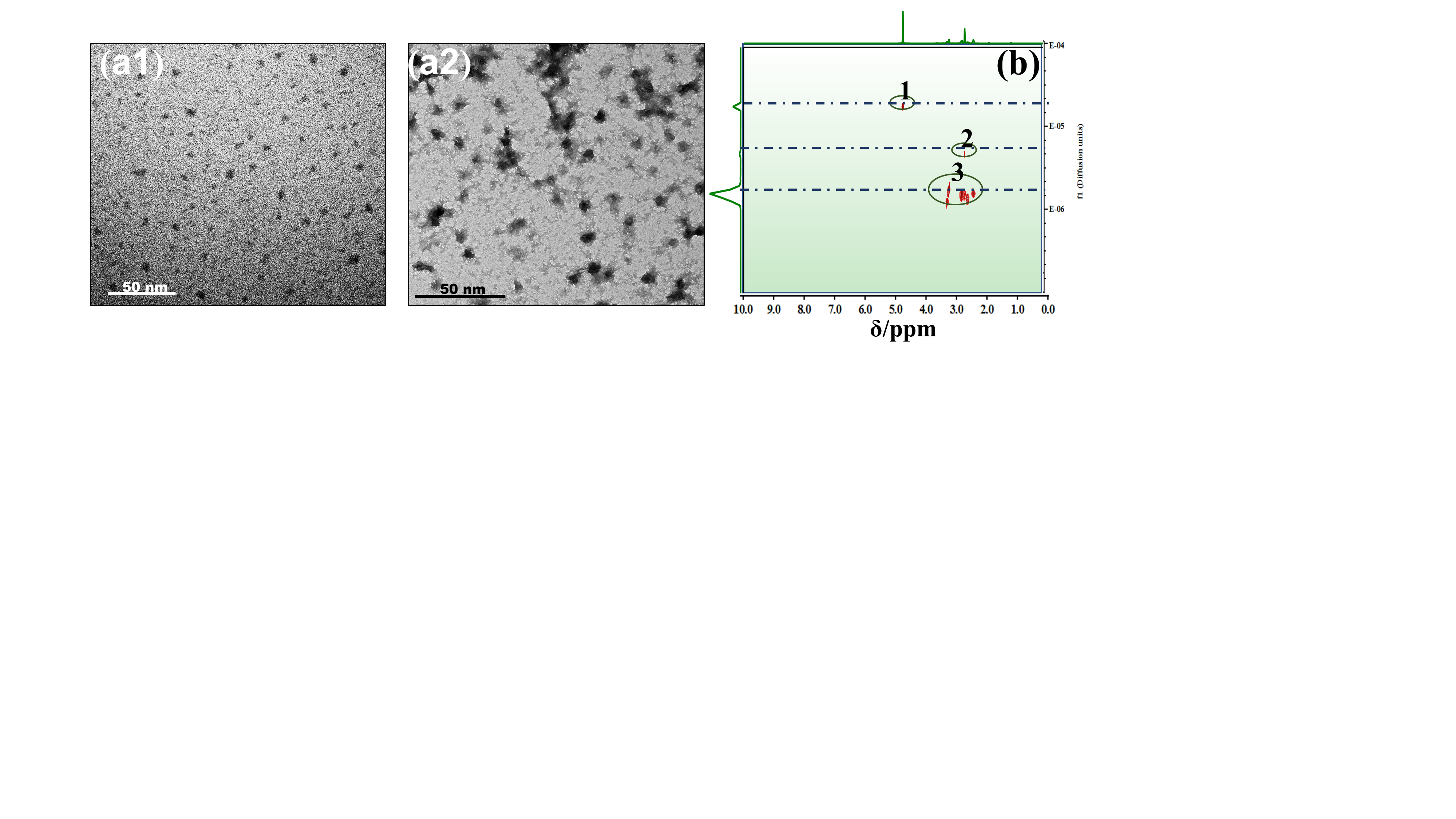


**Fig. S4 (a)** TEM images of G2 PAMAM, **(b)** 2D DOSY of G2 PAMAM


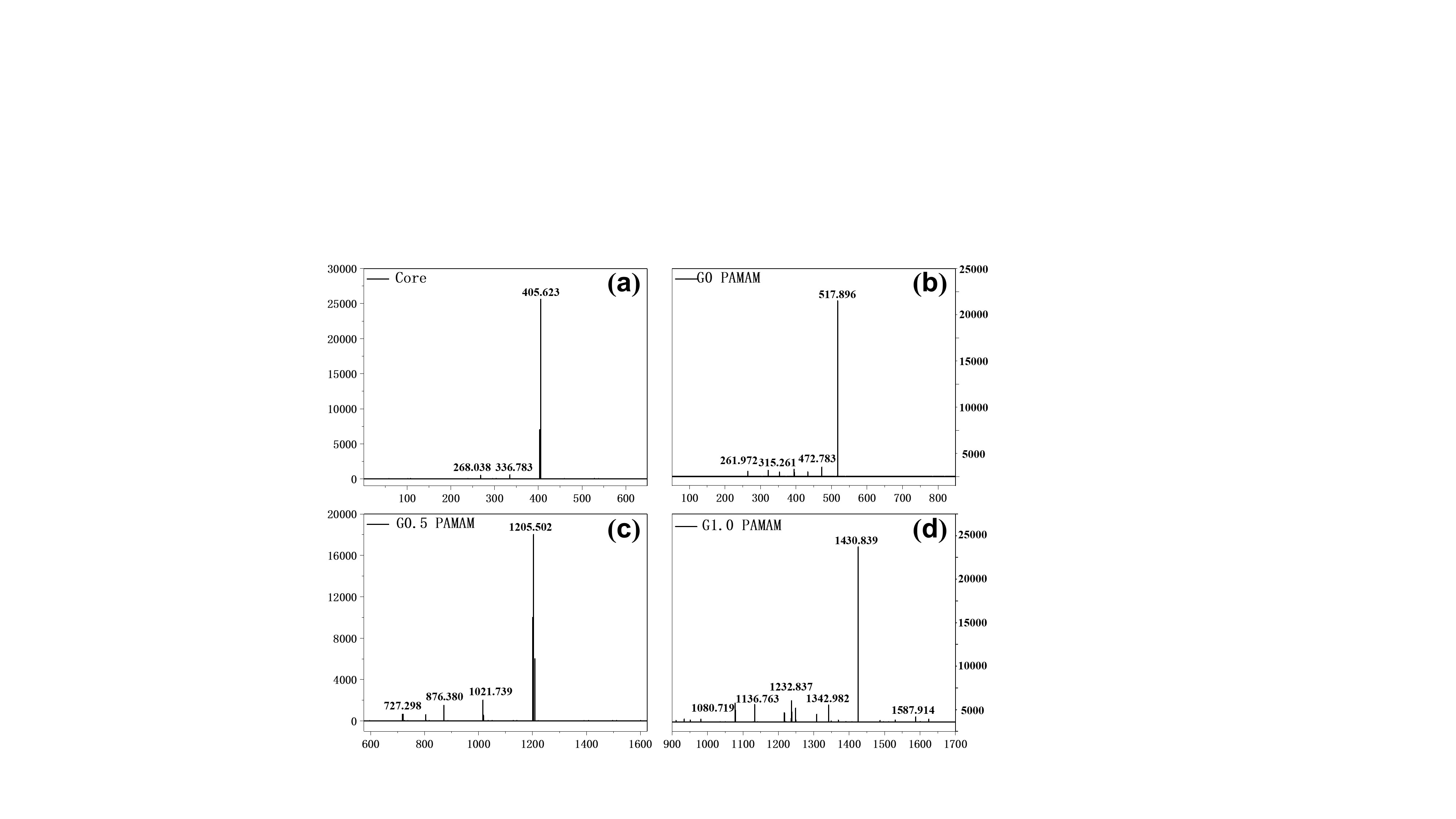


**Fig. S5 (a)** LC-MS of Core, **(b)** LC-MS of G0 PAMAM, **(c)** LC-MS of G0.5 PAMAM, **(d)** LC-MS of G1 PAMAM

**Fig. S6** Crosslinking density of CIIR/G2 PAMAM-H nanocomposites

Using the formula derived from the swelling equilibrium, n-octane is selected as the swelling solvent, and the interaction parameter between the solvent and the CIIR matrix
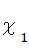
=0.52

The formula derived from the swelling balance was:


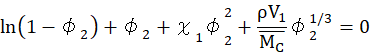


Where
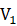
 was the partial molar volume of the solvent,
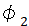
 was the volume fraction of CIIR in the swelling system,
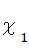
 was the interaction parameter between the solvent and CIIR,
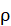
 was the solvent density and
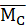
 was the average relative molecular mass between the crosslinking points, which characterizes the crosslink density of rubber.
